# Determination of *N*-nitrosodimethylamine in Ranitidine Dosage Forms by ESI-LC-MS/MS; Applications for Routine Laboratory Testing

**DOI:** 10.22037/ijpr.2021.115222.15258

**Published:** 2021

**Authors:** Jiao Liu, Zhi Zhao, Xiuling Yang, Yiran Jin, Xiujv Liu, Chuanping Wang, Zhiqing Zhang

**Affiliations:** *Department of Pharmacy, The Second Hospital of Hebei Medical University, Shijiazhuang, Hebei Province, China.*

**Keywords:** Valve switching technology, N-nitrosodimethylamine, Carcinogens, Ranitidine, High-performance liquid chromatography-mass spectrometry

## Abstract

A practical high-performance liquid chromatography-mass spectrometry method was developed for the analysis of *N*-nitrosodimethylamine (NDMA) characterized as an impurity, in combination with reports of the carcinogen found in ranitidine samples. After simple extraction of ranitidine samples, all compounds were analyzed with a high-performance liquid chromatography-mass spectrometry. Sensitivity was enhanced by employing the ten-way valve switching technology, which was examined to allow NDMA to enter the mass spectrometry and cut out the ranitidine samples extremely. A good linear relationship was observed within 3-100 ng·mL^−1 ^(r = 0.9992). The validated approach was effectively used to evaluate the NDMA contents in ranitidine samples in circulation, which revealed a difference among 21 batches. Quantitative determination of NDMA was within the scope of 3.38-57.05 ng·mL^-1^. Moreover, the contamination levels of NDMA in seven batches of products from six manufacturers were listed to exceed the acceptable daily intake. The sensitive method was verified to be appropriate to determine NDMA, even with low contents of NDMA in ranitidine products; the analysis of the selected samples reveals that some samples exceeded the national limit requirements. Therefore, it is worthwhile to conduct comprehensive quality control of the other drugs containing NDMA.

## Introduction

Nitrosamines are a group of carcinogens commonly found in pharmaceutical, food processing, and water treatment. Among them, *N*-nitrosodimethylamine (NDMA) is usually a genotoxic impurity nitrosamine compound, which belongs to class 2A carcinogen according to the classification of genotoxic impurities in ICH M7 general rules (1, 2). It can directly or indirectly damage DNA in cells, consequently enhancing the tendency of mutagenic and carcinogenic side effects. NDMA can be introduced through different ways, such as contaminated raw materials sourced from elsewhere, contaminated solvents and catalysts, or in the manufacturing process (3-4). Many products such as cured or smoked meats, dairy, vegetables, alcoholic beverages, and cereals are commonly found out the presence of NDMA contamination. Therefore, its risk of being carcinogenic has attracted much attention from the medical community in recent years (5).

**Figure 1:**
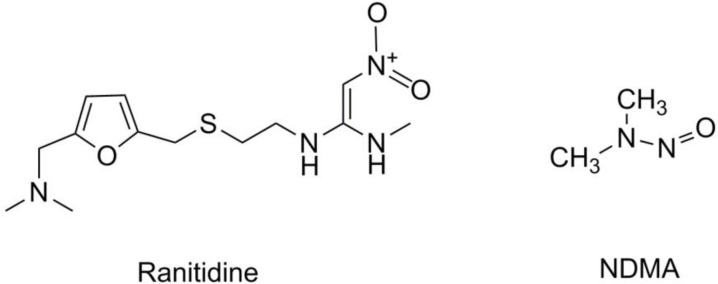
The structures of ranitidine and NDMA

Ranitidine which is widely used in treating gastric and duodenal ulcers, and gastroesophageal reflux disease (GERD) is a histamine H_2_-receptor antagonist (6, 7). Owing to the specific structure of ranitidine (Figure 1), NDMA is likely to emerge in the process of storage and transportation (8, 9). The new finding stated that the level of probable carcinogen NDMA in the drug was increased over time during storage based on its analysis of stockpiled ranitidine (10). Moreover, the amount of nitrite was also increased in connection with the storage of ranitidine drug substances and products at high temperatures, which was extrapolated as one factor influencing the formation of NDMA (11, 12). Exposing continuously to the high level of NDMA may increase the risk of cancer; it is urgent to focus on the quality and clinical safety of these drugs.

After certain products containing valsartan drugs were voluntarily recalled in many countries around the world owing to contamination with NDMA in July 2018 (13), the Food and Drug Administration (FDA) issued a statement that NDMA was also found in ranitidine drugs on September 13, 2019 (14). On April 1, 2020, the FDA issued a notice on its official website that unacceptably high levels of NDMA impurity were detected in some ranitidine drugs and claimed that ranitidine and its generic medicine should be removed and discarded from the pharmacy (10, 14). At the same time, the European Medicines Agency (EMA) required the suspension of all ranitidine medicines in Europe on April 30, 2020, due to the current risk of the use of ranitidine (15). On December 9, 2019, the Chinese Pharmacopoeia Committee proposed to revise the standards of ranitidine drugs and set out to draw up a document about NDMA impurity control. On May 8, 2020, China Medical Products Administration published the Technical Guidelines for The Study of Nitrosamines Impurities in Chemical Drugs (Trial), which regulated the limit of NDMA gene impurity of ranitidine in the market (16). Hence, an easy-to-perform, accurate and sensitive method for assessment of NDMA was developed to meet the requirement of the restricted control of NDMA.

There were various methods used for the determination of NDMA in complex substrates, such as gas chromatography (GC) (17), gas chromatography-mass spectrometry (GC-MS) (18-21), high performance liquid chromatography (HPLC) (13, 22, 23), high performance liquid chromatography-mass spectrometry (LC-MS/MS) (24, 25) and capillary electrophoresis (26). However, the current methods of analyzing NDMA in ranitidine samples reported were relatively rare and existed some limitations. Although two papers were demonstrated that NDMA in the ranitidine was determined by GC-MS combining with solid-phase extraction or microextraction, they had to be adopted a complex, cumbersome pre-treatment process (21, 27). More importantly, since the phenomenon that ranitidine could produce extra NDMA under heating conditions might cause the inaccuracy of results, GC or GC-MS, which were applied successfully in drugs such as sartan and metformin, should be avoided in the determination of the NDMA in the ranitidine. Therefore, HPLC or LC-MS/MS was an option for ranitidine samples. In our preliminary work, it failed to assay the NDMA accurately by HPLC owing to its shortage of sensitivity. And the conventional LC-MS/MS method could pollute and shorten the service life of the mass spectrum once a plug of sample solution was injected. As an effective shunt device, the switch valve had gained much attention since it sent the analyst, not a non-target compound, into the instrument. Taking this purpose into account, a single LC-MS/MS analysis method using a ten-way switch valve between the HPLC and mass spectrometer that permitted to separate and quantify NDMA content of ranitidine in currency was proposed in this manuscript so as to provide data for the clinical safety application of ranitidine. The method provided a sensitive, rapid and feasible approach for quality control of other drugs in terms of the content of the NDMA.

## Experimental

*Apparatus*

All experiments were operated on a Shimadzu HPLC system, including a binary gradient pump (LC-20AD), an autosampler (SIL-20AC), and a column oven (CTO-20A) (Shimadzu, Tokyo, Japan); SCIEX API 4000 triple quadruple mass spectrometer (Concord, Ontario, Canada) fitted with a heated nebulizer interface (APCI). A ten-way switching valve, item number: EPC10W, was manufactured by Valco Instruments Co., Inc., Houston, Texas, USA. It was used between HPLC and mass spectrometer for allowing NDMA to enter the mass spectrometry and cutting out the ranitidine samples extremely. Data acquisition was supported with Analyst software.

*Chemicals and Reagents*

NDMA (Chemical purity: 97.2%, the National Institutes for Food and Drug Control). Reference of ranitidine hydrochloride (chemical purity: 99.9%, Shanghai Yuanye Biotechnology Co. Ltd.).

A total of seventeen batches of ranitidine hydrochloride capsules were offered from nine manufacturers (manufactory A-manufactory I). Ranitidine citrate capsule was produced by one manufacturer (manufactory J). There were three batches of ranitidine hydrochloride injection produced by one manufacturer (manufactory K). The batch numbers of products provided by each manufactory are listed in Table 1.

Methanol (Seymour Fisher Technology Co. Ltd.). Deionized water (Watsons Co. Ltd.). Formic acid (Shanghai Aladdin Biotechnology Technology Co. Ltd.).

*Methods*

*Preparation of standard solutions*

Standard solutions were prepared by dissolving NDMA and ranitidine hydrochloride in methanol at a concentration of 1 μg·mL^-1^ and 1 mg·mL^-1^, respectively. The mixed standards were diluted with methanol at a stock concentration. All solutions were stored at 4 °C before analysis. The supernatant was transferred and filtered by 0.22 μm nylon syringe filtration before injection. The preparation and treating process of solutions involving NDMA was carried out in a fume hood since NDMA was a carcinogenic substance.

*Preparation of sample solutions*

*Preparation of ranitidine capsules*

The appropriate numbers of ranitidine hydrochloride capsules and ranitidine citrate capsules were respectively crushed to obtain a target weight of 300 mg and 200 mg of the active pharmaceutical ingredient into the 10 mL volumetric flask. Each batch of the sample was suspended in the appropriate volume of methanol (10 mL), mixed for about a minute using a vortex mixer, and extracted in an ultrasonic bath for 40 min. The sample was centrifuged for 10 min at 4500 rpm. Liquid supernatant was transferred and filtered by 0.22 μm nylon syringe filtration, then discarded the first 1 mL prior to injection.

*Preparation of ranitidine injections*

Batches of ranitidine injections were diluted independently to a target concentration of the methanol. The rest of the experiment operation was as mentioned above.

*Preparation of quality control solutions*

Quality control solutions of NDMA were obtained from dissolving appropriate mixed standard solutions with methanol at low, medium, and high concentration levels.

*Mass spectrometry conditions*

**Figure 2:**
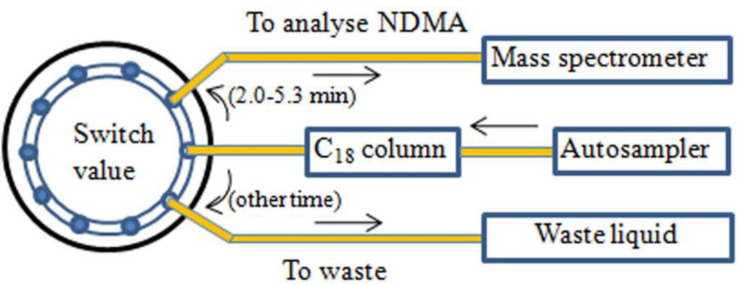
The design drawing for the circuit diagram

An APCI with the positive ion mode was performed in the mass spectrometer. The curtain gas, collision gas, nebulizer current, and temperature were set as the optimized main parameters at 15 psi, 11 psi, 5 μA, and 450 °C, respectively. The multiple reaction monitoring (MRM) was performed for quantification (Table 2). Particularly worth mentioning is the ten-way valve switching technique. It was programmed to permit the mobile phase with a retention time of 2-5.3 min to enter the mass spectrometer and the mobile phase with other retention time to enter the waste liquid. The design drawing for the circuit diagram is found in Figure 2.

*Chromatographic conditions*

Formic acid-methanol (A, 0.1%) and formic acid-water (B, 0.1%) as mobile phase were used to analyze the compounds on a Diamonsil C_18_ column (4.6 mm × 150 mm, 5 µm) with a flow rate of 1.2 mL·min^−1^. The gradient program was as follows: 2% A (0–2 min), 2–20% A (2–5 min), 20–100% A (5–6 min), 100% A (6–10 min). The equilibration time was required for 5 min before each injection. The column temperature was 45 °C, and the injection volume was 10 μL.

*Method validation*

*Specificity*

The selectivity was verified in comparison with the MRM chromatographic profiles of the blank methanol, NDMA standard solution, and free ranitidine standard solution before and after spiking with the NDMA to detect the interference of ranitidine to NDMA.

*Linearity, LOD and LOQ*

The calibration curve was performed by diluting standard solutions of NDMA to reflect the linearity, which range of concentrations were 3, 6, 10, 20, 50, 75, and 100 ng·mL^-1^. The determination coefficient of the calibration curve should be ≥ 0.999 to make the acceptable linearity meet the requirement. The limit of detection (LOD) and limit of quantification (LOQ) were defined when the signal-to-noise ratio was separately greater than 3 and 10.

*Accuracy and precision*

The index of precision and accuracy was assessed as relative standard deviation (RSD), and the percent ratios of the concentration were calculated in accordance with peak area to nominal concentration. Six replicates of each constitute at three set experimental concentrations were performed within one day and over three successive days to evaluate the intra-day and inter-day precisions, respectively. The range of accuracy should be 85.0% to 115.0%.

*Stability*

The stability described as peak area was estimated according to inject repetitively of quality control samples at three typical assay concentrations for 24 h. All solutions were kept at 4 °C.

*Recoveries*

The recoveries of NDMA were determined by measuring samples with the evaluation of precision and accuracy. The calibration graph was used to calculate the recoveries of NDMA, which was drawn from mixing in an equivalent amount of the standard solution of NDMA to the sample. The values of recovery ranged from 85.0% to 115.0%.

## Results

*Validation results*

*Specificity*

**Figure 3:**
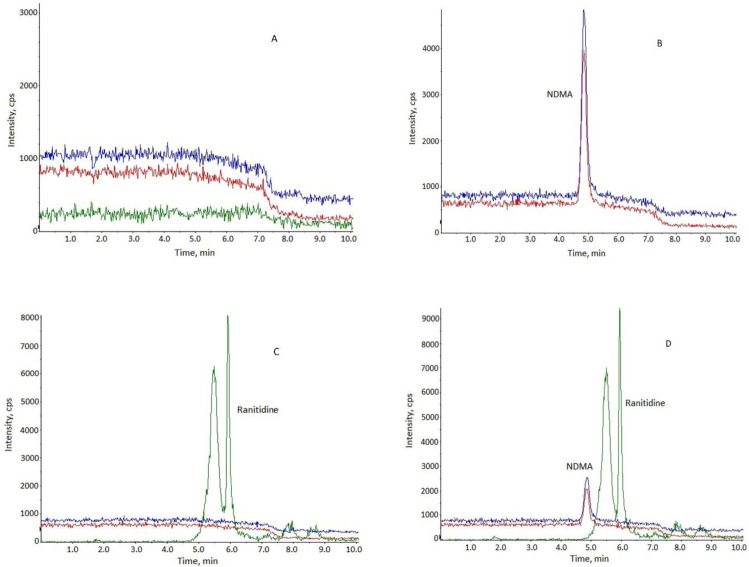
Representative chromatograms. (A) blank methanol; (B) NDMA standard solution; (C) ranitidine standard solution; (D) ranitidine solution with NDMA

The selectivity was performed by analyzing the NDMA, NDMA added into the ranitidine at the concentration of appropriate concentration. As shown in Figure 3, interference peaks were not coeluted at the retention time of NDMA.

*Linearity, LOD and LOQ*

Taking the concentration in ng/mL as abscissa (x) and the ratio of peak area as ordinate (y), the calibration graphs of NDMA was established. The straight line of NDMA acquired by six experiments with different concentrations had a good correlation coefficient. As the concentration range was 3-100 ng·mL^-1^, the calibration curve and its correlation coefficients for NDMA was y = 2670x + 0.0732 (R^2 ^= 0.9992). The LOD and the LOQ of NDMA were 1.0 ng·mL^-1^ and 3 ng·mL^-1^, respectively.

*Accuracy and precision*

Table 3 showed the intra- and inter-day accuracies of NDMA were in the range of 94.7%-102.0% with the RSDs below 4.9%, which demonstrated the reproducible and precise of the present method.

*Stability*

As listed in Table 3, the accuracies of NDMA were within the range of 97.2%-103.0%, and the RSDs were below 5.0%. The result confirmed that NDMA was observed to be stable for 24 h at 4 °C.

*Recoveries*

The results of recoveries of NDMA at three typical assay concentrations presented in Table 3 were within the range of 96.2%-103.0%, and the RSDs were below 4.7%.

*Sample analysis*

The applicability of this method to analyze NDMA in 21 batches of ranitidine products produced by 11 manufacturers in the market with the above-mentioned conditions was investigated. As mentioned in the Technical Guidelines for The Study of Nitrosamines Impurities in Chemical Drugs (Trial), different ranitidine products, including ranitidine hydrochloride capsule, ranitidine citrate capsule, and ranitidine injection, exist diversity in the national limit requirements (17). Table 4 summarized the relationship between the maximum daily dose of active pharmaceutical ingredients and the acceptable daily intake of NDMA in the respective ranitidine products.

When a sample solution of ranitidine was analyzed, the peak at 4.78 min was identified as NDMA peak in comparison to its retention time with that of the NDMA reference standard. And the results of the ranitidine products exposed in NDMA contamination are presented in Table 1. Quantitative determination of NDMA was within the scope of 3.38-57.05 ng·mL^-1^, which revealed a huge difference with regard to NDMA content of each batch. Among them, the extent of NDMA contamination of seven batches from six manufacturers was illustrated above the acceptable daily intake. The batch numbers were E190903, 1907012, 2002512, 190603, 200404, 1904221, and 42003120, respectively.

In addition, NDMA contents in the batch of E200304, E200307, 2005748, and 42003120 could be quantified and were indicated to be below the maximum daily limit of NDMA (<0.32 ppm for ranitidine hydrochloride capsule and <0.064 ppm for ranitidine citrate capsule). These NDMA contents, including batch numbers 200306, 1912409, 2004756, and 200501, were not quantified even though a small signal was acquired at the match position in the chromatogram. Thus, the rest of the batches of ranitidine products were considerably not detected in the active pharmaceutical ingredient.

## Discussion

*Selection of the ion source and application of switch value*

As a powerful analytical technique, HPLC-MS/MS has gained much attention in separation science (24, 25). Although the electrospray ionization (ESI) source was very common and sensitive for multi-compounds analysis, it was not considered as an ion source of suitable choice because of the unavailable sensitivity that is relative to small molecular weight of NDMA (M = 74.08). Analysis of NDMA applied with APCI source was intended to achieve high sensitivity rather than ESI source (28). The working principle of APCI was transferring the ions generated under atmospheric pressure to the non-ionizable compounds (such as NDMA) through electric dizzy needle discharge, thus producing molecular ions. In light of these, APCI was selected as the ion source to assay NDMA in this experiment.

To our knowledge, the main component of ranitidine drug and NDMA was easy elution together due to the similar polarity. The content determination of NDMA has significant effects on samples and external factors, which was attributed to the plenty of adjuvant in the sample and extreme content of the main component. Thus, the bigger resolution between the NDMA and drug ingredients was aspired to meet the high response of NDMA to be far away from the impact of main components (29).

In our preliminary experiment, the reproducibility and sensitivity of the determination of NDMA in the complex sample entered into the mass spectrometer were barely satisfactory. However, a ten-way switching valve device applied prior to mass spectrometry was carried out to let the flow of NDMA in and cut out samples as much as possible, hence protecting the mass spectrometry from contamination of samples and improving sensitivity. The reason was likely that when a plug of a sample solution containing drug substances and excipients was dynamically injected into the analytical instrument, the resolution of the column rapidly deteriorates, and interference peaks may occur in the retention time of NDMA so as to affect the production of molecular ions.

*Optimization of mass spectrum conditions*

In the optimization process, it was primarily to ensure the highest response and sensitivity of NDMA. The appropriate ion pair was discovered by the positive ion scanning mode. The mass spectrum system was optimized by adjusting the ion source parameters as well as MRM to achieve the ideal detection state (Table 2).

*Optimization of chromatographic conditions*

Compared to the extremely small content of NDMA, relatively large contents of main components and excipients entering the mass spectrometry could not merely interfere with the determination of trace NDMA to cause serious matrix effects but also pollute ion sources and reduce the service life of mass spectrometry. To make the trace NDMA meet the detection requirements, a ten-way switching valve device cutting unconnected compounds out of the mass spectrometry was recommended to get the optimal separation and high sensitivity. In the preliminary experiment, a phenomenon that the peak of NDMA could not decompose and overlap with other compounds was achieved, and the most significant variables influencing the performance of NDMA and other ingredients in ranitidine drugs were formic acid concentration, flow rate, and injection volume. As discussed above, the improved sensitivity and shortened migration time were obtained with the satisfactory mobile phase (0.1% formic acid-water and 0.1% formic acid-methanol) in gradient elution combined with optimized flow rate (1.2 mL·min^−1^) and 10 μL injection.

*Quantification of NDMA in samples*

Since unacceptable levels of nitrosamine impurities, including NDMA in the antihypertensive and anti-heart failure medicines known as angiotensin II receptor blockers (ARBs) were discovered by the FDA in 2018, the ARBs have been recommended multiple recalls. Further investigation has revealed that some ranitidine products, such as the commonly called brand-name drug Zantac also contained the NDMA impurity at low levels (30). In accordance with the carcinogens catalog issued by the World Health Organization, NDMA belongs to class 2A carcinogens (31). In order to ensure the safety and quality control of drugs and realize effective risk control, this technical guideline is formulated to provide guidance for the research and control of nitrosamine impurities in registered drugs and listed chemicals.

**Figure 4:**
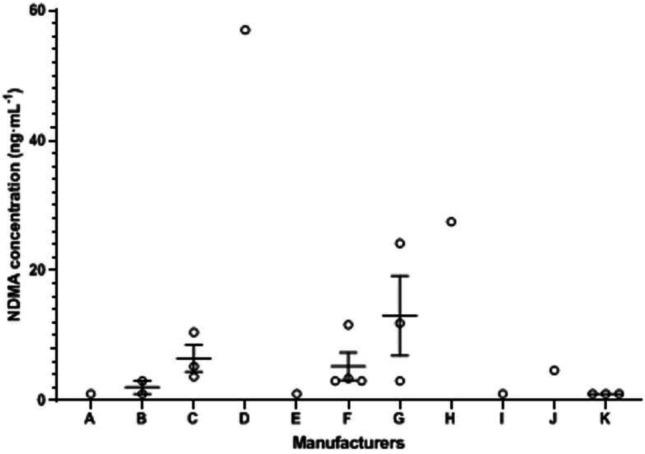
The correlation between the NDMA concentration and manufacturers

The correlation between the NDMA concentration and manufacturers is described in Figure 4. Results illustrated that commercial ranitidine products in 21 batches coming from different manufactories had exposed NDMA as pollution, and there was a big difference in the NDMA content of each batch which was speculated to various factors, such that the dispersion was considerably the consequence of different production technologies or follow-up storage conditions in the sale. In our research, the data from the content of NDMA above acceptable intake across 7 batches of ranitidine products probably existed a relationship with the date of manufacture. As can be seen from the batch number, most products produced earlier were more likely tend to exceed the maximum limit requirement of NDMA. The reason for this phenomenon may be that the related guiding principle of carcinogens was not be published by the statement so that the manufacturer did not pay enough attention to the NDMA in the early stage, or that the original level of NDMA in the drug below the regulation of limit gradually raised to the unacceptable scope over time during storage. In addition, the size of the company was subjectively considered one of the factors affecting the NDMA content. Furthermore, we hoped that existing samples stored one year later under the suitable condition would be measured again so as to discuss the influence of storage time.

Blue line: m/z 75.1/43.1; red line: m/z 75.1/58.1; green line: m/z 315.0/176.0.

**Table 1:** Overview on the concentrations of NDMA in 21 batches of ranitidine products

**Products**	**manufacturers**	**Specifications**	**Batches**	**concentrations (ng·mL** ^-1^ **)**	**concentrations (ppm)**
ranitidine hydrochloride capsule^a^	A	0.15 g × 30	306200201	n.d.	n.d.
	B	0.15 g × 30	200306	n.q.	n.q.
		0.15 g × 20	200321	n.d.	n.d.
	C	0.15 g × 30	E190903	10.49	0.35
			E200304	5.24	0.17
			E200307	3.67	0.12
	D	0.15 g × 30	1907012	57.05	1.90
	E	0.15 g × 30	20200202	n.d.	n.d.
	F	0.15 g × 30	1912409	n.q.	n.q.
		0.15 g × 20	2002512	11.65	0.39
			2004756	n.q.	n.q.
			2005784	3.38	0.11
	G	0.15 g × 30	190603	24.20	0.81
			200404	11.89	0.40
			200501	n.q.	n.q.
	H	0.15 g × 30	1904221	27.52	0.92
	I	0.15 g × 30	200303	n.d.	n.d.
ranitidine citrate capsule^b^	J	0.2 g × 14	42003120	4.64	0.23
ranitidine injection^c^	K	50 mg:2mL	19110602	n.d.	n.d.
			20021901	n.d.	n.d.
			20050701	n.d.	n.d.

n.d.: not detected (<1.0 ng·mL^-1^).n.q.: is not quantified (<3.0 ng·mL^-1^).

^a^acceptable daily intake of 0.32 ppm.

^b^acceptable daily intake of 0.064 ppm.

^c^acceptable daily intake of 0.48 ppm.

**Table 2:** The MRM parameters of NDMA and ranitidine

**Compounds**	**Q**_1_	**Q**_3_	**Dwell Time (msecond)**	**CE (V)**	**DP (V)**	**EP (V)**	**CXP (V)**	**Retention time (min)**
NDMA	75.1	43.1	500	23	70	10	11	4.78
NDMA	75.1	58.1	500	19	70	10	11	4.78
Ranitidine	315.0	176.0	100	25	60	10	11	-

**Table 3:** Intra-day and inter-day precision, stability, and recovery of NDMA (n = 6).

**Concentration** **(ng·mL** ^-1^ **)**	**Intra-day**		**Inter-day**		**Stability**		**Recovery**	
	**Precision (%)**	**RSD (%)**	**Precision (%)**	**RSD (%)**	**Accuracy (%)**	**RSD (%)**	**Accuracy (%)**	**RSD (%)**
6.0	94.7	4.6	98.6	4.9	97.2	0.4	96.6	2.1
20	101.0	1.7	102.0	1.9	95.5	2.6	96.2	1.6
100	102.0	2.1	102.0	1.8	103.0	5.0	103.0	4.7

**Table 4:** Interim limits for NDMA in ranitidine products expressed as ng/day and ppm

**Drugs**	**Maximum daily dose (mg/day)**	**Acceptable intake NDMA (ng/day)**	**Acceptable intake NDMA (ppm)**
ranitidine hydrochloride capsule	300	96	0.32
ranitidine citrate capsule	1500	96	0.064
ranitidine injection	200	96	0.48

## Conclusion

A practical HPLC-MS/MS method for quality control of ranitidine products by the rapid screen and evaluation of NDMA characterized as impurity has been developed combined with ten-way valve switching technology. Employing a ten-way switching valve device with phase switching was intended to enhance assay sensitivity for the quantitative analysis of NDMA in ranitidine-containing products. Thus, the applicability of this method to analyze NDMA in 21 batches of ranitidine products with optimized conditions was investigated. All of the results were verified that the suggested method was reliable and available to evaluate the quality of ranitidine products in terms of the trace concentration of NDMA, which was determined in contrast to the acceptable limit. And it has proved to be a powerful tool for ensuring the safety of the clinical medication. Furthermore, the application of the presented approach is expected to provide technical support for the quality control of other drugs contaminated by carcinogens like NDMA.

## Conflicts of interest

The authors declare that there are no conflicts of interest.

## References

[1] ICH M7 (2017). Assessment and control of DNA reactive (mutagenic) impurities in pharmaceuticals to limit potential carcinogenic risk. International Council for Harmonisation of Technical Requirements for Pharmaceuticals for Human Use (ICH).

[2] Zhang LY, Liu XL, Guo TS (2020). Analysis on control of genotoxic impurities in drug research. Chem. Ind..

[3] White CM (2020). Understanding and preventing (N-nitrosodimethylamine) NDMA contamination of medications. Ann. Pharmacother..

[4] Roux JL, Gallard H, Croué JP, Papot S, Deborde M (2012). NDMA formation by chloramination of ranitidine: kinetics and mechanism. Environ. Sci. Technol..

[5] Eads AV (2020). Pharmacists are the key in interpreting clinical implications of N-nitrosodimethylamine contamination in medications. J. Am. Pharm. Assoc..

[6] Kong N (2020). A comparative study of the clinical efficacy of omeprazole and ranitidine in the treatment of peptic ulcer. Guide. Chin. Med..

[7] Zeng T, Mitch WA (2016). Oral intake of ranitidine increases urinary excretion of N-nitrosodimethylamine. Carcinogenesis.

[8] Lv J, Wang L, Li YM (2017). Characterization of N-nitrosodimethylamine formation from the ozonation of ranitidine. J. Environ. Sci..

[9] Shaik KM, Sarmah B, Wadekar GS, Kumar P (2020). Regulatory updates and analytical methodologies for nitrosamine impurities detection in sartans, ranitidine, nizatidine, and metformin along with sample preparation techniques. Crit. Rev. Anal. Chem..

[10] Dyer O (2020). All ranitidine should be discarded, says US drug agency. BMJ..

[11] Dong C (2020). The potential safety risks of the common gastric drug: ranitidine. J. Health Times.

[12] Abe Y, Yamamoto E, Yoshida H, Usui A, Tomita N, Kanno H, Masada S, Yokoo H, Tsuji G, Uchiyama N, Hakamatsuka T, Demizu Y, Izutsu K, Goda Y, Okuda H (2020). Temperature-dependent formation of N-nitrosodimethylamine during the storage of ranitidine reagent powders and tablets. Chem. Pharm. Bull..

[13] Masada S, Tsuji G, Arai R, Uchiyama N, Demizu Y, Tsutsumi T, Abe Y, Akiyama H, Hakamatsuka T, Izutsu K, Goda Y, Okuda H (2019). Rapid and efficient high performance liquid chromatography analysis of N-nitrosodimethylamine impurity in valsartan drug substance and its Products. Sci. Rep..

[14] Xia XM (2020). An immediate withdrawal of all ranitidine products were ordered by the FDA. China. Pharm. Univ..

[15] Ryan ME, Barker C, Hawcutt DB (2020). Ranitidine in short supply: why now, and where next. Arch. Dis. Child..

[16] The China Center for Drug Evaluation Technical guidelines for the study of nitrosamines impurities in chemical drugs (Trial).

[17] Parr MK, Joseph JF (2019). NDMA impurity in valsartan and other pharmaceutical products: Analytical methods for the determination of N-nitrosamines. J. Pharm. Biomed. Anal..

[18] Tsutsumi T, Akiyama H, Demizu Y, Uchiyama N, Masada S, Tsuji G, Arai R, Abe Y, Hakamatsuka T, Izutsu K, Goda Y, Okuda H (2019). Analysis of an impurity, N-nitrosodimethylamine, in valsartan dug substances and associated products using GC-MS. Biol. Pharm. Bull..

[19] Shen CY, Cai HQ, Pei SF, Zhang Y (2019). Determination of nine nitrosamines in water by solid phase extraction and gas chromatography mass spectrometry. J. Occup. Environ. Med..

[20] Liu H, Zhang YC, Lu YF, Shi J, Gu WB (2017). Determination of N-nitrosodimethylamine in tobacco by gas chromatography-tandem mass spectrometry. Physical. J. Anal. Test..

[21] Lim HH, Oh YS, Shin HS (2020). Determination of N-nitrosodimethylamine in drug substances and products of sartans, metformin and ranitidine by precipitation and solid phase extraction and gas chromatography-tandem mass spectrometry. J. Pharm. Biomed. Anal..

[22] Fan TT, Zhang XD (2019). Determination of N-nitrosodimethylamine in valsartan by high performance liquid chromatography. J. Nutr. Med..

[23] Li WX, Chen N, Zhao YG, Guo WQ, Muhammd N, Zhu Y, Huang ZP (2018). Online coupling of tandem liquid-phase extraction with HPLC-UV for the determination of trace N-nitrosamines in food products. Anal. Methods..

[24] Sherf-Clavel O, Kinzig M, Besa A, Schreiber A, Bidmon C, Abdel-Tawab M, Wohlfart J, Sörgel F, Holzgrabe U (2019). The contamination of valsartan and other sartans, Part 2: Untargeted screening reveals contamination with amides additionally to known nitrosamine impurities. J. Pharm. Biomed. Anal..

[25] Zhang WW, Zhao CH, Fu M, Sun Q (2019). Determination of N-nitrosodimethylamine in food by HPLC-MS/MS with steam distillation separation. J. Anal. Test..

[26] Řemínek R, Foret F, Chung DS (2021). Application of capillary electrophoresis-nano-electrospray ionization-mass spectrometry for the determination of N-nitrosodimethylamine in pharmaceuticals. Electrophoresis..

[27] Giménez-Campillo C, Pastor-Belda M, Campillo N, Hernández-Córdoba M, Viñas P (2021). Development of a new methodology for the determination of N-nitrosamines impurities in ranitidine pharmaceuticals using microextraction and gas chromatography-mass spectrometry. Talanta.

[28] Sörgel F, Kinzig M, Abdel-Tawab M, Bidmon C, Schreiber A, Ermel S, Wohlfart J, Besa A, Sherf-Clavel O, Holzgrabe U (2019). The contamination of valsartan and other sartans, part 1: New findings. J. Pharm. Biomed. Anal..

[29] Guo CC, Liu Q, Zhang L, Zheng J, Wang Y, Yang SJ, Chu ZJ, Niu C, Xu YW (2020). Determination of N-nitrosodimethylamine in metformin hydrochloride and its preparations by high performance liquid chromatography-tandem mass spectrometry. Chromatography.

[30] The Food and Drug Administration Zantac (ranitidine): Safety information – NDMA found in samples of some ranitidine medicines. The U. S. Food and Drug Administration. [online] 2019 Sep 13 [cited 2020 Jun 15]. 2019 Sep 13.

[31] The National Medical Products China Food and Drug Administration WHO international agency for research on cancer list of carcinogens. [online] 2017 Qct 30 [cited 2020 Jun 15]. [serial online].

